# FOXO4 as a Redox-Sensitive Regulator of Antioxidant Defense and Cellular Senescence: Cysteine-Based Signaling, p53 Interaction, and Therapeutic Targeting

**DOI:** 10.3390/antiox15070842

**Published:** 2026-07-03

**Authors:** Diana-Maria Mateescu, Dragos-Mihai Gavrilescu, Adelina-Raluca Marinescu, Ovidiu Rosca, Voichita Elena Lazureanu, Adrian-Cosmin Ilie, Camelia-Oana Muresan, Alexandra Enache

**Affiliations:** 1 Department of General Medicine, Doctoral School, “Victor Babes” University of Medicine and Pharmacy, Eftimie Murgu Square 2, 300041 Timisoara, Romania; diana.mateescu@umft.ro; 2 Department of Orthodontics, Dental District, Zăgazului 3, One Floreasca Vista, Sector 1, 014261 Bucharest, Romania; dr.gavrilescu@outlook.com; 3 Discipline of Infectious Diseases, Department XIII, “Victor Babes” University of Medicine and Pharmacy, Eftimie Murgu Square 2, 300041 Timisoara, Romania; adelina.marinescu@umft.ro (A.-R.M.); ovidiu.rosca@umft.ro (O.R.); lazureanu.voichita@umft.ro (V.E.L.); 4 Department of Public Health and Sanitary Management, “Victor Babes” University of Medicine and Pharmacy, Eftimie Murgu Square 2, 300041 Timisoara, Romania; 5 Centre for Translational Research and Systems Medicine, “Victor Babes” University of Medicine and Pharmacy, Eftimie Murgu Square 2, 300041 Timisoara, Romania; 6 Legal Medicine, Timisoara Institute of Legal Medicine, 300041 Timisoara, Romania; enache.alexandra@umft.ro; 7 Ethics and Human Identification Research Center, “Victor Babes” University of Medicine and Pharmacy, Eftimie Murgu Square 2, 300041 Timisoara, Romania; 8 Discipline of Forensic Medicine, Bioethics, Deontology, and Medical Law, Department of Neuroscience, “Victor Babes” University of Medicine and Pharmacy, Eftimie Murgu Square 2, 300041 Timisoara, Romania

**Keywords:** FOXO4, forkhead transcription factors, reactive oxygen species, redox signaling, oxidative stress, antioxidant defense, cellular senescence, p53, FOXO4-DRI, NRF2, aging

## Abstract

(1) Background: Reactive oxygen species (ROS) act as physiological signaling mediators but contribute to oxidative damage, cellular dysfunction, and age-related disease when redox homeostasis fails. Forkhead box O4 (FOXO4) has emerged as a redox-sensitive regulator linking stress adaptation, antioxidant defense, and cellular senescence. This structured narrative review critically evaluates which redox- and aging-related conclusions are supported directly for FOXO4 and which remain inferred from other FOXO isoforms. (2) Methods: PubMed/MEDLINE, Scopus, and Web of Science were searched from inception to May 2026; Google Scholar was used only for supplementary citation tracking and did not contribute a separate platform-level count. Of 420 records, 300 remained after deduplication, 110 full texts were assessed, and 89 publications were retained. FOXO4-related evidence was classified as directly FOXO4-specific (*n* = 18), FOXO-family/conserved (*n* = 24), or extrapolated predominantly from FOXO1/FOXO3/DAF-16 (*n* = 20); 27 contextual publications on redox biology, senescence, disease, and NRF2 were tracked separately. (3) Results: The strongest FOXO4-specific evidence supports three mechanistic axes: cysteine-dependent redox sensing, stress-regulated nuclear trafficking and coactivator engagement through transportin-1 and p300/CBP, and FOXO4–p53-mediated survival of senescent cells. By contrast, direct FOXO4 regulation of commonly cited antioxidant targets, including SOD2, catalase, sestrins, and GADD45, remains insufficiently demonstrated and is inferred mainly from FOXO3 or broader FOXO-family studies. FOXO4-DRI has shown senolytic activity in preclinical models, including vascular endothelium, but has not been clinically validated. (4) Conclusions: FOXO4 is a redox-responsive transcriptional regulator with well-supported roles in cysteine-based signaling and senescent-cell survival, whereas its target-gene-level antioxidant program remains incompletely resolved. Clinical translation of FOXO4–p53 disruption requires isoform- and tissue-specific validation, pharmacokinetic and delivery studies, long-term toxicology, and explicit assessment of p53-dependent tumor surveillance.

## 1. Introduction

Reactive oxygen species (ROS) occupy a paradoxical position in cell biology. At low, tightly controlled fluxes, they act as second messengers that modulate signal transduction. However, when production overruns antioxidant capacity, they damage lipids, proteins, and nucleic acids, contributing to cancer, metabolic disease, neurodegeneration, and aging [1,2]. The principal intracellular sources of ROS—the mitochondrial electron transport chain and the NADPH oxidase (NOX) family—generate superoxide and hydrogen peroxide. Their subcellular localization confers specificity to redox signaling, particularly through reversible oxidation of susceptible protein cysteine thiols [3,4,5]. Cells therefore depend on transcriptional programs that calibrate antioxidant capacity to the prevailing redox state. Among the regulators that fulfill this role, the forkhead box O (FOXO) family has emerged as a conserved hub linking nutrient availability, growth-factor signaling, oxidative-stress responses, and cell-fate decisions.

The mammalian FOXO subfamily comprises four members: FOXO1, FOXO3, FOXO4, and FOXO6. All share a conserved winged-helix forkhead DNA-binding domain and recognize the DAF-16 family member-binding element (DBE) [6,7,8,9]. These proteins show partial functional redundancy but also distinct tissue- and stimulus-specific roles. FOXO4 is expressed mainly in muscle, kidney, and colorectal tissue [9,10]. FOXO3 is well-characterized as a regulator of antioxidant gene expression and longevity; FOXO3 variants are among the strongest human longevity associations [11,12,13,14,15,16]. FOXO4 (also known as AFX), present at modest levels in many normal tissues, has received less attention yet remains a uniquely informative model for investigating how FOXO activity is controlled by the cellular redox state [17,18,19,20].

Two developments have brought FOXO4 to the foreground of redox and aging biology. First, biochemical work showed that FOXO4 is not merely a passive target of upstream kinases. It is governed directly by thiol–disulfide chemistry, providing one of the clearest examples of a transcription factor acting as a redox sensor [19,21]. Second, the discovery that FOXO4 maintains the viability of senescent cells by retaining p53 in the nucleus identified the FOXO4–p53 interface as a tractable therapeutic target. This finding motivated the design of the senolytic peptide FOXO4-DRI [22]. Both themes are intimately connected to oxidative stress: ROS both activate FOXO4 and accumulate in senescent cells. FOXO4 is therefore of direct interest to readers concerned with antioxidants and redox homeostasis.

This review integrates structural, biochemical, cellular, and translational evidence on FOXO4. It focuses on three questions: how FOXO4 senses oxidative signals, which antioxidant outputs are directly attributable to FOXO4 rather than other FOXO isoforms, and whether the FOXO4–p53 interaction can be targeted safely in senescent cells. Evidence is graded explicitly throughout the text.

The FOXO4-centered perspective is important because most antioxidant target-gene data derive from FOXO3 or broader FOXO-family studies. The review therefore separates direct FOXO4-specific findings from conserved-family evidence, extrapolation, and contextual literature, with particular emphasis on cysteine-based redox sensing, senescent-cell survival, vascular senescence, and the still poorly defined relationship between FOXO4 and NRF2–KEAP1 signaling.

## 2. Methods: Structured Narrative Review Approach

This article was designed as a structured narrative review with mechanistic and translational aims. It was not conducted as a formal systematic review or meta-analysis; however, the search domains, eligibility criteria, screening procedure, extraction fields, and evidence-classification rules were predefined to improve transparency and limit isoform-level overinterpretation.

### 2.1. Search Strategy and Information Sources

PubMed/MEDLINE, Scopus, and Web of Science were searched from database inception to May 2026. Google Scholar was used only for backward and forward citation tracking and verification of potentially relevant records; its unstable platform-level result count was not included in the numerical flow. Search terms covered FOXO4/AFX, oxidative stress, redox signaling, antioxidant defense, cysteine oxidation, senescence, p53, FOXO4-DRI, senolytics, and NRF2–KEAP1 signaling. The core search string was as follows:

(“FOXO4” OR “AFX” OR “forkhead box O4”) AND (“redox” OR “oxidative stress” OR “reactive oxygen species” OR “antioxidant defense” OR “cysteine oxidation” OR “thiol oxidation”).

Targeted searches combined FOXO4 with p53, senescence, FOXO4-DRI/D-retro-inverso, transportin-1, p300/CBP, and NRF2/KEAP1. Reference lists of key primary mechanistic and structural articles were screened manually. Potentially eligible citations identified through citation tracking were checked against the consolidated database export before inclusion.

### 2.2. Eligibility Criteria

Studies were eligible for inclusion if they met at least one of the following criteria: (i) they investigated FOXO4/AFX directly in relation to redox signaling, oxidative stress, antioxidant defense, cysteine-dependent regulation, senescence, p53 interaction, or pharmacological targeting; (ii) they examined conserved FOXO-family mechanisms relevant to redox biology and included FOXO4 among the investigated isoforms; or (iii) they provided mechanistic insight into NRF2–KEAP1 signaling relevant to comparison with FOXO4-based redox regulation.

Studies focused exclusively on FOXO1, FOXO3, or FOXO6 were included only when they clarified conserved FOXO-family mechanisms directly relevant to antioxidant defense, stress adaptation, or redox-dependent transcriptional regulation. Exclusion criteria were non-biomedical commentaries, non-peer-reviewed sources, studies without mechanistic relevance to FOXO4/redox biology, articles focused solely on unrelated FOXO functions, and studies in which FOXO4 was mentioned only incidentally without experimental or conceptual relevance.

### 2.3. Study Selection and Data Extraction

Two authors (D.-M.M. and D.-M.G.) independently screened titles and abstracts, assessed potentially eligible full texts, and extracted the study design, biological model, FOXO isoform, redox mechanism, post-translational modification, target genes or pathways, senescence/p53 relationship, pharmacological intervention, and translational relevance. Disagreements were resolved by consensus after re-examination of the original article. Primary FOXO4-specific research was prioritized over secondary summaries whenever a mechanistic claim was made.

The consolidated search yielded 420 records. After removal of 120 duplicates, 300 unique records underwent title/abstract screening; 190 were excluded as outside the review questions. Of 110 full texts assessed, 21 were excluded because FOXO4 was only incidental, the article addressed unrelated FOXO functions, or it added no relevant mechanistic or translational information. The final qualitative synthesis included 89 publications. The complete numerical flow and exclusion summary are provided in Table S1.

Among the 89 retained publications, 18 provided direct FOXO4-specific evidence, 24 addressed FOXO-family or conserved mechanisms that included FOXO4, and 20 were extrapolated predominantly from FOXO1, FOXO3, or DAF-16. A further 27 contextual publications informed redox biology, senescence, disease, clinical senolysis, or NRF2 comparison but were not used to establish FOXO4-specific causality. Reference-level classification is provided in Table S2, and the primary sources supporting the key FOXO4–redox and FOXO4–p53 claims are summarized in Table S3.

### 2.4. Evidence Classification and Strength of Inference

FOXO4-related findings were assigned to three evidentiary levels (Table 1). Direct evidence required experimental, structural, genetic, or pharmacological testing of FOXO4 itself. FOXO-family evidence included conserved mechanisms or multi-isoform studies in which FOXO4 was examined but not isolated as the sole causal factor. Extrapolated evidence derived mainly from FOXO1, FOXO3, or DAF-16 and was considered hypothesis-generating for FOXO4. Contextual publications were tracked separately and were not used to infer FOXO4-specific regulation.

Only direct FOXO4-specific evidence was considered sufficient for isoform-specific mechanistic conclusions. FOXO-family evidence was treated as supportive but non-exclusive, whereas extrapolated evidence was presented using cautious language such as “may participate,” “is consistent with,” or “requires FOXO4-specific validation.”

## 3. Structural and Molecular Features of FOXO4

**Figure 1:**
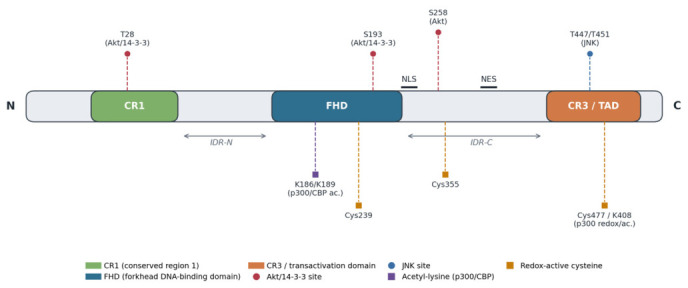
Schematic domain architecture of human FOXO4 and selected regulatory residues. The folded forkhead DNA-binding domain (FHD) is flanked by intrinsically disordered regions (IDR-N, IDR-C) containing CR1 and CR3 (transactivation domain). Approximate positions are shown for Akt/14-3-3 phosphorylation sites, the stress-activated JNK sites (Thr447/Thr451), acetyl-lysines targeted by p300/CBP, and redox-active cysteines (Cys239, Cys355, Cys477). NLS, nuclear localization sequence; NES, nuclear export sequence.

FOXO4 has the modular architecture typical of FOXO proteins (Figure 1). Its central forkhead domain (FHD), the principal folded region, is flanked by intrinsically disordered N- and C-terminal regions [23,24]. The original high-resolution NMR structure of the human AFX/FOXO4 DNA-binding domain established the canonical winged-helix fold, comprising three α-helices, three β-strands, and two characteristic wings [23]. Short conserved elements within the disordered regions include CR1 near the N-terminus and CR3, the main C-terminal transactivation domain (TAD) [24,25].

The FHD recognizes the consensus DBE sequence 5′-GTAAACAA-3′ and the related insulin-responsive element; unlike several other forkhead proteins, the FOXO4 FHD binds DNA as a monomer [25]. An important regulatory feature is the interplay between the disordered TAD and the FHD: NMR studies indicate that CR3 transiently contacts the DNA-binding surface of the FHD and contributes to discrimination between target and non-target DNA, a minor conformational state that effectively masks the non-target-binding interface and refines specificity beyond what the FHD achieves alone [25,26]. The protein also contains a nuclear localization sequence (NLS) overlapping the C-terminal part of the FHD and a nuclear export sequence (NES), the balance of which sets subcellular distribution; 14-3-3 binding to the phosphorylated NLS region sterically masks it and shifts FOXO4 to the cytoplasm [27].

Because most of FOXO4 outside the FHD is intrinsically disordered, the protein offers numerous low-complexity surfaces for post-translational modification and for transient, multivalent interactions with partners such as 14-3-3, p300/CBP, transportin-1, β-catenin, and p53 [6,19,21,27,28,29]. This conformational plasticity is increasingly viewed as functionally central: it allows a single polypeptide to integrate multiple inputs and to switch rapidly between cytoprotective and pro-apoptotic outputs [6,30]. Table 2 summarizes the principal functional elements of FOXO4 and selected key residues discussed throughout this review.

## 4. Upstream Regulation and the Post-Translational Code

### 4.1. The PI3K–Akt–14-3-3 Axis

The best-characterized control of FOXO4 is exerted by the phosphatidylinositol 3-kinase (PI3K)–Akt pathway downstream of insulin and growth factors. Akt phosphorylates FOXO4 at three conserved Ser/Thr residues; phosphorylation at the first two creates docking motifs for 14-3-3 proteins, which bind FOXO4, mask its NLS and obscure the DNA-binding surface, and shift the equilibrium toward cytoplasmic localization and inactivation [27,31,33,35]. Direct control of the FOXO4 orthologue AFX by protein kinase B (Akt) was among the founding observations of this regulatory paradigm [33,36]. Under growth-favorable conditions FOXO4 transcriptional activity is thus suppressed; withdrawal of growth-factor signaling or PI3K inhibition reactivates it, permitting nuclear re-entry and target-gene expression [6,32]. This axis is also the principal conduit of insulin action, embedding FOXO4 within metabolic control [37,38].

### 4.2. Stress-Activated Kinases: Ral–JNK, p38, and AMPK

Oxidative stress engages a distinct and, in important respects, opposing layer of regulation. In contrast to insulin signaling, low levels of hydrogen peroxide activate the small GTPase Ral, which triggers c-Jun N-terminal kinase (JNK)-dependent phosphorylation of FOXO4 at Thr447 and Thr451 within the C-terminal region; this promotes nuclear translocation and transcriptional activation and is also employed by tumor necrosis factor-α [34]. JNK-mediated activation of FOXO is evolutionarily conserved and re-deploys, under oxidative challenge, the same factor that anabolic signaling silences [17,34]. Additional stress kinases converge on FOXO factors: p38 promotes nuclear localization following DNA damage [39], and the energy sensor AMPK phosphorylates FOXO at distinct sites to enhance transcription of stress-resistance genes without altering localization [40]. These inputs collectively allow FOXO4 to function as a coincidence detector of nutrient status, energy state, and redox stress [6,41].

### 4.3. Acetylation, Deacetylation, Ubiquitination, and Methylation

Beyond phosphorylation, FOXO4 activity is shaped by reversible acetylation. The acetyltransferases p300 and CBP acetylate lysine residues of FOXO4, generally attenuating DNA binding and promoting nuclear export, whereas the NAD^+^-dependent deacetylase SIRT1 reverses these modifications and biases output toward stress resistance rather than apoptosis [42,43]. FOXO4 was shown to be acetylated upon peroxide stress and deacetylated by SIRT1, coupling its acetylation status to cellular energy and redox state [42]. Protein stability is governed by ubiquitination: in cells with active PI3K signaling, SKP2 mediates poly-ubiquitination and proteasomal degradation of FOXO, whereas MDM2 catalyzes mono- and poly-ubiquitination [44,45]. Notably, FOXO4 undergoes rapid, transient monoubiquitination after oxidative stress that increases its nuclear localization and activity, and the deubiquitinase USP7/HAUSP removes this mark—a regulatory loop strikingly parallel to that of p53 [44,46]. Finally, arginine methylation by PRMT1 at residues within the Akt consensus motif blocks Akt-mediated phosphorylation, thereby antagonizing nuclear exclusion and degradation [47]. Together these modifications constitute the combinatorial “FoxO code” that determines which subset of target genes is engaged in a given context [7,41]. Table 3 lists the principal modifications of FOXO4.

## 5. FOXO4 as a Direct Redox Sensor

A defining feature of FOXO4 is that its regulation by ROS is not solely indirect through stress kinases; it is also controlled by direct, reversible oxidation of specific cysteine thiols. This places FOXO4 among the relatively small set of transcription factors that act as bona fide redox sensors, alongside the cysteine-based sensing of KEAP1 in the NRF2 system [4,17,18,48].

Two complementary disulfide-based mechanisms have been defined. First, oxidation of Cys477 is required for hydrogen peroxide-induced acetylation of FOXO4 by p300/CBP: ROS promote a cysteine-thiol disulfide-dependent complex between FOXO and the p300/CBP acetyltransferase, and modulation of FOXO biological activity by p300/CBP-mediated acetylation depends fully on the formation of this redox-dependent complex [19]. The disulfide is subsequently reduced by the thioredoxin system, coupling FOXO4 modification to a major cellular antioxidant pathway [19]. Second, oxidation of Cys239 or Cys355 promotes an intermolecular disulfide bond between FOXO4 and the nuclear import receptor transportin-1 (TNPO1), which facilitates nuclear import and activation under oxidative stress; the FOXO orthologue DAF-16 in Caenorhabditis elegans uses the same TNPO1-dependent, disulfide-mediated import mechanism, highlighting deep evolutionary conservation [21].

**Figure 2:**
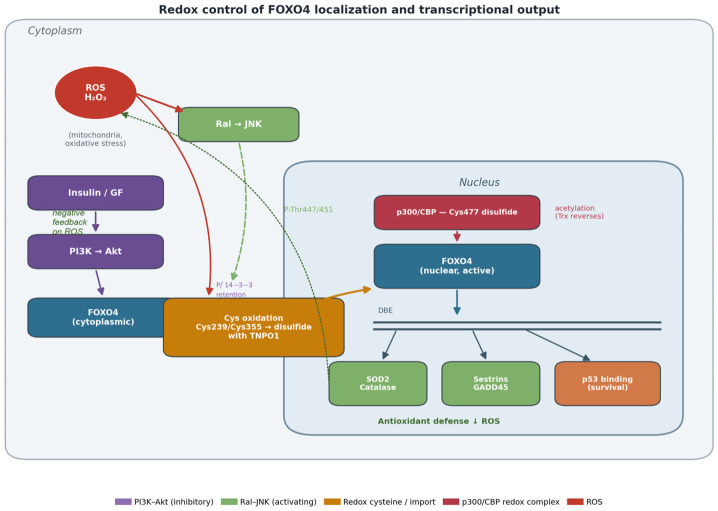
Redox control of FOXO4 localization and output. PI3K–Akt–14-3-3 signaling favors cytoplasmic retention, whereas Ral–JNK signaling and oxidation-dependent disulfides with transportin-1 and p300/CBP promote nuclear import or coactivator engagement. The transportin-1 and p300/CBP mechanisms are supported directly for FOXO4. Antioxidant targets shown downstream (SOD2, catalase, sestrins, and GADD45) represent conserved or extrapolated FOXO outputs and have not all been validated as direct FOXO4 targets. Solid arrows indicate direct or well-established directional relationships; dashed arrows indicate indirect or pathway-mediated regulation; and dotted arrows indicate proposed or context-dependent redox connections. Arrowheads indicate the direction of regulation.

These observations illustrate how distinct cysteines route the same oxidative signal toward different functional outcomes—one disulfide directing acetylation, another directing nuclear import—so that the pattern of cysteine oxidation effectively encodes information about the intensity and source of oxidative stress [17,19,21]. They also connect FOXO4 to upstream redox relays: peroxiredoxins and the thioredoxin system act as proximal sensors and transducers of peroxide, and earlier work linking forkhead regulation to a p66Shc-dependent pathway anticipated the concept of redox-controlled FOXO activity [2,4,18]. Importantly, the same H_2_O_2_ that activates FOXO can, via the insulin pathway, also transiently inactivate it—the “peroxide dilemma”—so that the net effect on FOXO localization depends on dose, kinetics, and cellular context [49]. Figure 2 integrates these inputs into a single regulatory scheme.

## 6. Transcriptional Output: FOXO4 and the Antioxidant Defense Program

Once active in the nucleus, FOXO factors orchestrate a transcriptional program spanning ROS detoxification, DNA repair, cell-cycle arrest, autophagy, and apoptosis [6,30,50]. The antioxidant arm is most relevant to redox homeostasis. The seminal demonstration that FOXO3 protects quiescent cells from oxidative stress by directly activating manganese superoxide dismutase (MnSOD/SOD2)—thereby lowering ROS—established the FOXO–antioxidant link and tied increased oxidative-stress resistance to longevity [11]. FOXO factors also induce catalase, which together with SOD2 reduces the steady-state burden of superoxide and hydrogen peroxide [11,17].

Additional FOXO targets contribute to redox balance and stress adaptation. The sestrins (including SESN3) are FOXO3-inducible antioxidant proteins that limit mitochondrial ROS, and GADD45 links FOXO activity to DNA-damage repair [51,52]. FOXO factors further regulate mitochondrial gene expression and metabolism, influencing the rate of ROS production at source rather than only scavenging downstream [53]. More recently, the FOXO target OSER1 (oxidative-stress-responsive serine-rich protein 1) was identified as an evolutionarily conserved effector whose expression extends lifespan and increases oxidative-stress resistance across species, expanding the known FOXO antioxidant repertoire [54].

Although these antioxidant targets are central to FOXO biology, the extent to which they are directly regulated by FOXO4 remains incompletely defined. SOD2, catalase, SESN3, GADD45, mitochondrial metabolic genes, and OSER1 have been characterized mainly in FOXO1/FOXO3 or broader FOXO-family contexts [11,17,51,52,53,54]. Therefore, their inclusion in this review should not be interpreted as definitive evidence of FOXO4-specific transcriptional regulation. Rather, these targets define a conserved FOXO antioxidant module in which FOXO4 involvement is biologically plausible because of the conserved DBE, partial functional redundancy among FOXO isoforms, and evidence from combined FOXO1/FOXO3/FOXO4 deletion models [6,55,56]. Direct FOXO4-specific validation, however, remains necessary using isoform-resolved chromatin profiling, transcriptomic analyses, and loss- or gain-of-function approaches. In addition, FOXO autoregulatory loops and β-catenin-dependent enhancement of FOXO antioxidant transcriptional activity suggest that FOXO4 may participate in broader stress-adaptive networks, although the isoform-specific contribution of FOXO4 within these pathways requires further clarification [29,55,57,58]. Representative redox-relevant FOXO target genes and the strength of evidence supporting FOXO4 involvement are summarized in Table 4.

### The “Peroxide Dilemma” and Context-Dependent Outputs

A recurring theme is that FOXO activation does not always lower ROS. Depending on context, FOXO factors can promote antioxidant defense or, conversely, drive pro-apoptotic and even pro-oxidant programs—the “peroxide dilemma” of insulin and redox signaling [49]. Whether FOXO4 ultimately protects a cell or commits it to death is set by the integrated pattern of post-translational modifications, the available coactivators and corepressors, the duration and intensity of the oxidative signal, and crosstalk with partners such as p53 and β-catenin [6,7,29,49]. This conditional logic underlies the apparently opposite roles of FOXO4 reported across tissues and disease states and is central to interpreting interventions that activate or inhibit it.

## 7. FOXO4 in Cellular Senescence and the FOXO4–p53 Axis

Cellular senescence—a stable cell-cycle arrest accompanied by a pro-inflammatory secretory phenotype (the senescence-associated secretory phenotype, SASP)—is both a consequence of oxidative and genotoxic stress and a driver of tissue aging; it is now recognized as a hallmark of aging and a therapeutic target in age-related disease [59,60,61,62,63]. A landmark study identified FOXO4 as a factor elevated in senescent cells that maintains their viability by interacting with p53: FOXO4 binds p53 and helps retain it in the nucleus, restraining the transcription-independent mitochondrial pro-apoptotic activity of p53 and thereby allowing senescent cells to resist death [22].

**Figure 3:**
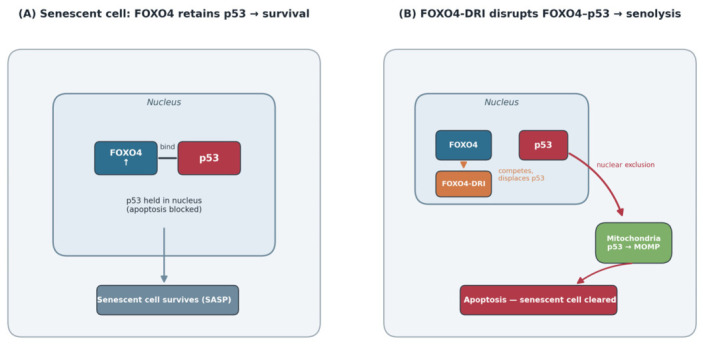
FOXO4–p53 signaling in senescence and its disruption by FOXO4-DRI. (**A**) Following oxidative or genotoxic stress, FOXO4 interacts with p53 and contributes to its nuclear retention, thereby limiting mitochondrial p53-mediated apoptosis and supporting senescent-cell survival. (**B**) FOXO4-DRI competitively disrupts the FOXO4–p53 interaction, promoting p53 nuclear exclusion and mitochondrial redistribution, mitochondrial outer membrane permeabilization (MOMP), and apoptosis of the senescent cell. This mechanism has been demonstrated in preclinical models. Blue indicates FOXO4 or FOXO4-associated elements, red indicates p53 and apoptosis-related processes, orange indicates FOXO4-DRI, green indicates mitochondrial p53 redistribution, and gray-blue indicates senescent-cell survival. Arrowheads indicate the direction of the interaction or cellular outcome. Light-blue shading identifies the nuclear compartment; the remaining background shading is used only for visual organization.

This insight motivated the design of FOXO4-DRI, a D-retro-inverso cell-penetrating peptide that mimics the FOXO4 region engaging p53 and competitively disrupts the FOXO4–p53 interaction [22]. By releasing p53, FOXO4-DRI promotes its nuclear exclusion, mitochondrial relocalization, and selective apoptosis (senolysis) of senescent cells. In mice, the peptide counteracted features of chemotoxicity-induced and natural aging and was comparatively well tolerated in the tested models [22]. Subsequent structural and biophysical studies refined this mechanism: solution NMR showed that disordered FOXO4-DRI binds the p53 transactivation domain, particularly p53 TAD2, in a transiently folded complex and that p53 phosphorylation increases affinity for both FOXO4 and FOXO4-DRI; complementary analyses showed that FOXO4 can engage both the transactivation and DNA-binding regions of p53 and inhibit p53–DNA binding [28,64]. Figure 3 contrasts the senescent-cell survival state with peptide-induced senolysis. Thus, the central FOXO4–p53 claims in this section are grounded in the original FOXO4-DRI study and subsequent primary structural and biophysical investigations rather than in secondary reviews [22,28,64].

The FOXO4–p53 axis links redox stress to senescent-cell persistence. FOXO4-DRI has produced senolytic effects in the original chemotoxicity and aging models and in senescent Leydig cells [22,65]. It should, however, be distinguished from other senolytics such as dasatinib plus quercetin (D + Q), navitoclax, and fisetin [66,67]. A phase 2 randomized clinical trial of intermittent D + Q therapy provided early human evidence regarding the feasibility and biological effects of senolytic treatment; however, these findings do not constitute clinical validation of FOXO4-DRI [68]. FOXO4-DRI has not been evaluated in human trials; its evidence base remains preclinical, and findings from D + Q cannot be transferred to a peptide that acts by disrupting FOXO4–p53 [22,64].

Accordingly, FOXO4–p53 disruption should be described as an emerging preclinical strategy rather than a clinically validated intervention. Tissue selectivity, immune effects, peptide delivery, bioavailability, pharmacokinetics, off-target toxicity, repeated-dose safety, and preservation of p53-dependent genomic and tumor surveillance remain unresolved [64,66].

## 8. FOXO4 in Oxidative-Stress-Related Disease

### 8.1. Cancer: Tumor Suppression and Context-Specific Roles

FOXO proteins are redox-responsive regulators of proliferation, apoptosis, and stress resistance and are generally regarded as tumor suppressors. Inactivation of FOXO downstream of constitutive PI3K–Akt signaling or through accelerated SKP2/MDM2-mediated degradation is common in cancer [8,45]. FOXO4 contributes to tumor suppression: in colorectal cancer, it is downregulated relative to normal tissue and restrains migration and metastasis through the APC2/β-catenin axis; under genotoxic stress, JNK-dependent activation enhances FOXO4 output [34,69]. Conversely, FOXO4-dependent survival of therapy-induced or otherwise senescent tumor cells could, in principle, create a senolytic vulnerability, but this application remains conceptual and requires tumor-specific validation [66,70]. This duality echoes the peroxide dilemma at the organismal scale.

### 8.2. Cardiovascular, Metabolic, and Reproductive Aging

In the cardiovascular system, FOXO factors influence cardiomyocyte growth and survival and can blunt cardiac hypertrophy through inhibition of calcineurin/NFAT signaling, an effect opposed by PI3K/Akt activity [9]. This cardiac interpretation is FOXO-family or extrapolated evidence rather than a FOXO4-specific conclusion. The PI3K–Akt–FOXO axis is also a principal conduit of insulin signaling, linking FOXO factors, including FOXO4, to metabolic disease [9,37,38]. Under oxidative stress, JNK-dependent FOXO activation can increase antioxidant transcriptional output [34]; however, any protective effect in type 2 diabetes remains predominantly a FOXO-family inference, with only partial direct evidence for FOXO4. In skeletal muscle, the FOXO1/FOXO3/FOXO4 network regulates both ubiquitin–proteasome and autophagy–lysosome pathways through targets such as atrogin-1, MuRF1, LC3, and BNIP3, linking FOXO signaling to muscle atrophy and sarcopenia during aging and catabolic stress [71,72,73]. This represents FOXO-family evidence in which FOXO4 is one of several contributing isoforms. By contrast, the senescence-linked role of FOXO4 in reproductive and other tissues is supported by direct FOXO4-specific preclinical evidence: disruption of FOXO4–p53 can clear senescent cells and restore selected tissue functions in experimental models [22,65]. Across these systems, whether FOXO4 is protective or deleterious depends on the magnitude and duration of oxidative stress, the tissue involved, and the integrated post-translational context [17,74].

### 8.3. Endothelial and Cerebrovascular Senescence

Endothelial senescence is a functional driver of vascular aging because it impairs nitric oxide signaling, redox balance, barrier integrity, microvascular density, and neurovascular coupling [75,76]. In aged mice, senescent endothelial-cell accumulation has been linked causally to blood–brain barrier disruption and microvascular dysfunction, while genetic or pharmacological senescence targeting improved selected vascular and neurovascular endpoints [76,77,78,79,80]. These studies establish senescence as a therapeutically actionable vascular process but, except for the FOXO4-targeted study described below, do not demonstrate a FOXO4-specific mechanism. Direct preclinical evidence was provided in senescent endothelial cells and naturally aged or D-galactose-treated mice: FOXO4-DRI reduced FOXO4–p53 binding, promoted p53 redistribution and apoptosis of senescent endothelial cells, lowered ROS and SASP markers, and improved aortic function [81]. This finding supports a FOXO4-specific role in vascular endothelium at the preclinical level; it does not establish efficacy in humans or a FOXO4-specific mechanism in human cerebrovascular aging.

Because several disease-related statements rest on FOXO-family inference rather than FOXO4-specific data, Table 5 grades the strength of FOXO4 evidence across the principal oxidative-stress-related disease contexts discussed above.

## 9. Pharmacological Modulation of FOXO4

Therapeutic modulation of FOXO4 follows two conceptually different directions. One seeks to enhance conserved FOXO stress-defense programs through upstream signaling or nuclear retention; evidence for a specifically FOXO4-mediated antioxidant benefit remains limited and context-dependent [49,58]. The other seeks to eliminate senescent cells by disrupting FOXO4–p53. FOXO4-DRI is the prototype, and primary structural studies now define the interaction surface more precisely [22,28,64]. The latter strategy has stronger FOXO4-specific preclinical support but also greater safety constraints because it intersects with p53 biology. Table 6 separates supported preclinical findings from uncertain and speculative translational claims [82].

No FOXO4-targeted intervention has yet demonstrated clinical efficacy. Rational development therefore requires peptide or small-molecule optimization, tissue-selective delivery, pharmacokinetic and pharmacodynamic characterization, repeated-dose toxicology, and direct assessment of p53-dependent tumor surveillance. Evidence from D + Q or other senolytics should be cited as support for the broader senolysis concept, not as validation of FOXO4-DRI.

## 10. FOXO4 and the NRF2–KEAP1 Axis: Two Redox-Responsive Strategies

It is useful to compare FOXO4 with the well-characterized antioxidant regulator NRF2. Both participate in redox-responsive transcriptional programs, but they sense and integrate oxidative signals through distinct mechanisms. NRF2 is constitutively synthesized and, under basal conditions, is recruited by the cysteine-rich adaptor KEAP1 to a CUL3-dependent ubiquitin ligase complex for proteasomal degradation. Oxidative or electrophilic modification of KEAP1 cysteines disrupts this process, allowing NRF2 to accumulate, translocate to the nucleus, heterodimerize with small Maf proteins, and activate antioxidant-response-element (ARE)-controlled genes, including enzymes involved in glutathione synthesis, NAD(P)H:quinone oxidoreductase 1, and heme oxygenase-1 [48,83,84]. Thus, KEAP1 is the principal redox sensor in this system, whereas NRF2 functions as the downstream transcriptional effector [48]. Table 7 compares the sensors, regulatory logic, biological outputs, and translational concerns of the two systems.

FOXO4 differs from NRF2 by combining redox sensing and transcriptional effector functions within one largely disordered protein. Its cysteines and redox-dependent interactions regulate nuclear import and coactivator recruitment [19,21], while its outputs can include stress adaptation, cell-cycle control, apoptosis, or senescent-cell survival [6,30,49]. NRF2–KEAP1 and FOXO4 are therefore complementary rather than interchangeable systems. In vascular aging, reduced NRF2-dependent stress resilience may favor endothelial senescence, whereas FOXO4 may support persistence of cells that have already become senescent [81,85]. This sequential relationship is mechanistically plausible but has not been tested directly; studies manipulating both pathways in the same isoform- and tissue-resolved models are needed.

Recent literature further highlights the need to interpret NRF2–KEAP1 signaling in a context-dependent manner. In aging biology, the NRF2–HO-1 axis is linked to oxidative-stress control, cellular senescence, SASP modulation, and inflammaging, highlighting the contribution of NRF2 decline or dysfunction to age-related inflammation [86]. In oncology, however, persistent KEAP1–NRF2 activation can confer tumor-cell survival, metabolic, and therapy-resistance advantages, illustrating the dual role of NRF2 as both a stress-defense regulator and a potential facilitator of cancer progression [87,88]. KEAP1 regulation also extends beyond cysteine oxidation: post-translational modifications including alkylation, glycosylation, glutathionylation, and S-sulfhydration can influence KEAP1–NRF2 binding, NRF2 stabilization, nuclear translocation, and antioxidant transcription [89]. Together, these findings reinforce the rationale for comparing NRF2–KEAP1 and FOXO4 as distinct but partially convergent redox-responsive regulatory systems.

## 11. Discussion and Open Questions

FOXO4 is not simply an interchangeable FOXO paralogue. Its best-supported distinguishing features are direct cysteine-dependent redox regulation and the FOXO4–p53 interaction that supports senescent-cell survival. By contrast, target-gene-level antioxidant claims remain dominated by FOXO3 or multi-isoform evidence. The central interpretive requirement is therefore to separate direct FOXO4 mechanisms from conserved-family biology and extrapolation.

FOXO4 integrates growth-factor, energy, and oxidative inputs through phosphorylation, acetylation, ubiquitination, methylation, and cysteine oxidation. These modifications can shift the protein between cytoprotective, cell-cycle, apoptotic, and senescence-supporting outputs. The resulting context dependence explains why broad FOXO4 activation or inhibition is unlikely to have uniform effects across tissues.

The main evidence gaps are as follows: direct identification of FOXO4-bound antioxidant genes; mapping of stimulus-specific FOXO4 cysteine oxidation states; clarification of FOXO4–NRF2 crosstalk; definition of tissue-specific functions in vascular, metabolic, muscular, and malignant contexts; and long-term evaluation of FOXO4–p53 disruption, including delivery, pharmacokinetics, immune effects, off-target toxicity, and tumor surveillance [19,21,64,66,81].

### Strengths and Limitations

Strengths of this review include its FOXO4-centered scope, prioritization of original mechanistic and structural studies, explicit evidence grading, and separate treatment of vascular senescence and translational safety. The search flow, reference-level classification, and primary-source map are provided in the Supplementary Materials.

The review also has limitations. It is a structured narrative synthesis rather than a systematic review or meta-analysis, and no formal risk-of-bias tool was applied. Selection bias cannot be excluded, experimental models are heterogeneous, and several disease and antioxidant claims depend on FOXO-family or FOXO3-derived evidence. FOXO4-DRI remains preclinical; therapeutic statements should therefore be interpreted as evidence-weighted hypotheses rather than clinical recommendations.

## 12. Conclusions and Future Directions

FOXO4 is a redox-responsive regulator that links reversible cysteine chemistry, stress-dependent trafficking, and senescent-cell survival. Direct evidence is strongest for disulfide-mediated interactions with transportin-1 and p300/CBP and for the FOXO4–p53 axis. Direct regulation of commonly cited antioxidant genes by FOXO4 remains incompletely demonstrated, and conclusions in this area should remain explicitly conditional.

Priority experiments include FOXO4-specific ChIP-seq or CUT&RUN under defined oxidative conditions; isoform-resolved transcriptomics and loss/rescue studies in senescent endothelial and other tissue-specific models; redox proteomics of Cys239, Cys355, and Cys477; and integrated studies of FOXO4 with NRF2–KEAP1. Before FOXO4–p53 disruption advances toward clinical testing, optimized delivery, pharmacokinetics, repeated-dose toxicology, tissue selectivity, immune consequences, and p53-dependent tumor surveillance must be established.

## Figures and Tables

**Table 1:** Evidence-classification framework used to interpret FOXO4-related findings.

Evidence Category	Definition	Interpretation in This Review
Direct FOXO4-specific	Experimental, genetic, structural, or pharmacological testing of FOXO4 itself	Supports FOXO4-specific conclusions
FOXO-family/conserved	Multi-isoform or conserved FOXO mechanisms that include FOXO4	Supportive; isoform specificity remains uncertain
Extrapolated	Evidence derived mainly from FOXO1, FOXO3, or DAF-16 without direct FOXO4 testing	Hypothesis-generating; requires FOXO4-specific validation
Contextual	Redox, senescence, disease, clinical senolysis, or NRF2 literature used for background/comparison	Not used to infer FOXO4-specific causality

**Table 2:** Functional elements and selected residues of human FOXO4.

Element	Description/Function	Selected Residues/Notes
Forkhead domain (FHD)	Winged-helix DNA-binding domain; only well-folded region; binds DBE as a monomer	Consensus 5′-GTAAACAA-3′ [23,25]
CR1	N-terminal conserved region within IDR-N; contains an Akt/14-3-3 motif	Akt site [31,32]
NLS/NES	Nuclear localization/export; NLS overlaps C-terminal FHD; masked by 14-3-3	14-3-3 binding [27]
CR3/TAD	C-terminal transactivation domain; transiently contacts FHD to tune DNA selectivity	Coactivator binding [25,26]
Akt/14-3-3 sites	Phosphorylation promotes 14-3-3 binding and cytoplasmic retention	Thr28, Ser193, Ser258 [31,32,33]
JNK sites	Stress-induced phosphorylation promoting nuclear import	Thr447, Thr451 [34]
Redox cysteines	Form intermolecular disulfides with TNPO1 and p300/CBP under oxidative stress	Cys239, Cys355, Cys477 [19,21]

**Table 3:** Post-translational and redox-dependent modifications regulating FOXO4 under physiological and oxidative-stress conditions.

Modification	Enzyme/Partner	Functional Consequence
Phosphorylation (Akt sites)	Akt/14-3-3	Cytoplasmic retention; inactivation [31,33,35]
Phosphorylation (Thr447/451)	Ral–JNK	Nuclear import; activation under oxidative stress [34]
Phosphorylation (stress/energy)	p38/AMPK	Nuclear localization; stress-gene induction [39,40]
Acetylation	p300/CBP	Reduced DNA binding; nuclear export [19,42]
Deacetylation	SIRT1 (NAD^+^)	Bias toward stress-resistance program [42,43]
Mono-/poly-ubiquitination	MDM2/SKP2 (USP7 reverses)	Nuclear import (mono) or degradation (poly) [44,45,46]
Arginine methylation	PRMT1	Blocks Akt phosphorylation; stabilizes nuclear FOXO [47]
Disulfide with TNPO1	Transportin-1 (Cys239/Cys355)	Facilitated nuclear import; activation [21]
Disulfide with p300/CBP	p300/CBP (Cys477)	Enables ROS-induced acetylation; reversed by thioredoxin [19]

**Table 4:** Representative redox-relevant FOXO target genes and the strength of evidence supporting FOXO4 involvement.

Target	Category	Role in Redox Homeostasis/Stress Response	Supporting Evidence	FOXO4-Specificity
SOD2/MnSOD	Antioxidant enzyme	Catalyzes mitochondrial superoxide dismutation and lowers intracellular ROS burden	Directly demonstrated mainly in FOXO3-dependent antioxidant responses [11]	Extrapolated
Catalase	Antioxidant enzyme	Detoxifies hydrogen peroxide and contributes to cellular peroxide buffering together with SOD2	Supported in broader FOXO antioxidant programs [11,17]	FOXO-family
Sestrins, including SESN3	Antioxidant/metabolic signaling	Limit mitochondrial ROS and interact with stress-response and mTOR-related pathways	Characterized mainly as FOXO3-inducible antioxidant targets [52]	Extrapolated
GADD45	DNA repair/cell-cycle arrest	Links FOXO activation to DNA-damage repair, stress adaptation, and cell-cycle control	Characterized mainly in FOXO3 or broader FOXO contexts [51]	Extrapolated
OSER1	Oxidative-stress-responsive protein	Conserved FOXO target associated with lifespan extension and increased oxidative-stress resistance	Identified as an evolutionarily conserved FOXO-regulated stress-response effector [54]	FOXO-family/extrapolated
Mitochondrial metabolic genes	Metabolic regulation	Modulate mitochondrial function and may reduce ROS production at source rather than only enhancing ROS scavenging	Supported mainly by FOXO3-derived evidence on mitochondrial gene regulation [53]	Extrapolated
FOXO-dependent antioxidant program in hematopoietic stem cells	Integrated antioxidant network	Maintains physiological ROS detoxification and limits apoptosis in vivo	Combined FOXO1/FOXO3/FOXO4 deletion increases ROS and apoptosis, indicating a role for endogenous FOXO factors including FOXO4 [56]	FOXO-family
β-catenin-enhanced FOXO antioxidant targets	Redox-responsive transcriptional cooperation	β-catenin binds FOXO factors and enhances antioxidant transcriptional output under oxidative stress	Supported by FOXO-family and model-organism evidence, including sod-3 regulation [29,55,57,58]	FOXO-family/extrapolated

Evidence categories follow Table 1. “Direct” denotes FOXO4-specific testing; “FOXO-family” denotes multi-isoform or conserved evidence including FOXO4; “Extrapolated” denotes evidence derived mainly from other FOXO isoforms and requiring direct FOXO4 validation.

**Table 5:** Evidence strength for FOXO4 involvement across oxidative-stress-related disease contexts. Grading follows the framework in Table 1.

System/Disease Context	Principal FOXO4-Related Role	Supporting Evidence	Evidence Strength for FOXO4
Cancer (tumor suppression)	Context-dependent tumor suppression; FOXO4 downregulated in colorectal cancer and restrains migration/metastasis via APC2/β-catenin; JNK-dependent activation under genotoxic stress	Direct FOXO4 study in colorectal cancer [69]; FOXO-family tumor-suppressor data [8,45,67]	Direct (colorectal)/FOXO-family (general)
Cancer (senescent-cell-like tumors)	Proposed senolytic disruption of FOXO4–p53 in mutant-p53 contexts	Preclinical/conceptual extension of FOXO4-DRI senolysis [22,66]	Preclinical/speculative
Cardiac remodeling	FOXO factors blunt hypertrophy via calcineurin/NFAT inhibition; relieved by PI3K/Akt	FOXO-family cardiac data; FOXO4 inferred [9]	FOXO-family/extrapolated
Metabolic disease (type 2 diabetes)	PI3K–Akt–FOXO axis links FOXO4 to insulin signaling; JNK-mediated activation can raise antioxidant gene expression	FOXO-family and insulin-signaling data; partial FOXO4 involvement [9,34,37,38]	FOXO-family/extrapolated
Skeletal muscle atrophy/sarcopenia	FOXO1/3/4 network drives ubiquitin–proteasome and autophagy programs (atrogin-1, MuRF1, LC3, BNIP3)	FOXO-family network data including FOXO4 [71,72,73]	FOXO-family
Reproductive/tissue aging	FOXO4–p53 maintains senescent-cell survival; FOXO4-DRI clears senescent cells and restores function	Direct FOXO4-DRI studies in senescence models [22,65]	Direct (preclinical)
Endothelial and cerebrovascular senescence	FOXO4–p53 supports senescent endothelial-cell survival; FOXO4-DRI improves vascular function (preclinical)	Direct endothelial FOXO4-DRI study [81]; broader cerebrovascular senescence studies [75,76,77,78]	Direct preclinical (endothelium); contextual (cerebrovascular)

**Table 6:** Evidence-based interpretation of FOXO4 as a therapeutic target.

Claim	Current Evidence	Interpretation	Translational Status
FOXO4 is elevated in senescent cells	Preclinical cellular and animal data showing increased FOXO4 expression or functional relevance in senescent-cell survival [22,81]	Supported	Mechanistically relevant
FOXO4–p53 disruption induces senolysis	FOXO4-DRI studies and primary structural/biophysical analyses of FOXO4–p53 disruption [22,28,64,81]	Supported preclinically	Not clinically validated
FOXO4 activation improves antioxidant defense	Mostly FOXO-family or FOXO3-derived evidence involving antioxidant targets such as SOD2, catalase, SESN3, GADD45, OSER1, and mitochondrial stress-response genes [11,17,51,52,53,54,55,56]	Plausible but not definitively FOXO4-specific	Requires isoform-specific testing
FOXO4 targeting improves human healthspan	No direct clinical proof; current evidence derives from experimental senescence, aging, and senolysis models [22,65,67,81]	Speculative	Not established
Long-term FOXO4–p53 targeting is safe	Insufficient long-term in vivo and human data; concerns include p53-dependent tumor surveillance, tissue specificity, delivery, and off-target toxicity [64,66,81]	Major unresolved issue	Requires toxicology, pharmacokinetic, tissue-selectivity, and tumor-surveillance studies

**Table 7:** Comparative redox-sensing strategies and biological outputs of the NRF2–KEAP1 and FOXO4 systems.

Feature	NRF2–KEAP1	FOXO4
Primary redox sensor	KEAP1 cysteines	FOXO4 cysteines and partner-protein disulfides
Main transcriptional output	Detoxification and antioxidant gene expression	Antioxidant defense, cell-cycle arrest, autophagy, apoptosis, and senescent-cell survival
Core regulatory mechanism	Oxidative or electrophilic modification of KEAP1 cysteines stabilizes NRF2 and promotes its nuclear accumulation	PI3K–Akt inhibition, stress-activated JNK signaling, acetylation, ubiquitination, methylation, and cysteine oxidation regulate FOXO4 localization and activity
Biological logic	Predominantly cytoprotective and detoxification-oriented	Context-dependent; cytoprotective, stress-adaptive, pro-apoptotic, or senescence-supporting depending on cellular context
Relationship to aging	NRF2 decline or dysregulation contributes to impaired stress resistance, inflammaging, and age-related disease	FOXO4 links oxidative-stress responses with cellular senescence and senescent-cell persistence through the FOXO4–p53 axis
Cancer-related concern	Persistent NRF2 activation may support tumor-cell survival, therapy resistance, and metabolic adaptation	Disruption of FOXO4–p53 may affect p53-dependent tumor surveillance and requires careful long-term safety evaluation
Translational implication	Pharmacological NRF2 modulation requires balancing cytoprotection against possible cancer-promoting effects	FOXO4 targeting requires isoform-specific, tissue-specific, and p53-safety-focused validation

## Data Availability

No new data were generated or analyzed in this study. All information is derived from previously published studies, which are cited within the manuscript.

## Supplementary Materials

The following supporting information can be downloaded at https://www.mdpi.com/article/10.3390/antiox15070842/s1. Table S1: Literature search and selection flow; Table S2: Reference-level evidence classification; Table S3: Primary sources supporting key FOXO4-specific and translational claims.

## Abbreviations

The following abbreviations are used in this manuscript:

AFX	Alternative historical name for FOXO4
AMPK	AMP-activated protein kinase
APC2	Adenomatous polyposis coli 2
ARE	Antioxidant-response element
CBP	CREB-binding protein
CR1	Conserved region 1
CR3	Conserved region 3
CUL3	Cullin 3
D + Q	Dasatinib plus quercetin
DAF-16	Caenorhabditis elegans FOXO orthologue
DBE	DAF-16 family member-binding element
DNA	Deoxyribonucleic acid
FHD	Forkhead domain
FOXO	Forkhead box O
FOXO1	Forkhead box O1
FOXO3	Forkhead box O3
FOXO4	Forkhead box O4
FOXO4-DRI	FOXO4 D-retro-inverso peptide
FOXO6	Forkhead box O6
GADD45	Growth arrest and DNA damage-inducible 45
H_2_O_2_	Hydrogen peroxide
IDR	Intrinsically disordered region
IDR-C	C-terminal intrinsically disordered region
IDR-N	N-terminal intrinsically disordered region
JNK	c-Jun N-terminal kinase
KEAP1	Kelch-like ECH-associated protein 1
LC3	Microtubule-associated protein 1 light chain 3
MDM2	Mouse double minute 2 homolog
MnSOD	Manganese superoxide dismutase
MOMP	Mitochondrial outer membrane permeabilization
MuRF1	Muscle RING finger 1
NAD^+^	Nicotinamide adenine dinucleotide
NADPH	Nicotinamide adenine dinucleotide phosphate
NES	Nuclear export sequence
NFAT	Nuclear factor of activated T cells
NLS	Nuclear localization sequence
NMR	Nuclear magnetic resonance
NOX	NADPH oxidase
NRF2	Nuclear factor erythroid 2-related factor 2
NQO1	NAD(P)H:quinone oxidoreductase 1
OSER1	Oxidative-stress-responsive serine-rich protein 1
p53	Tumor protein p53
PI3K	Phosphatidylinositol 3-kinase
PRMT1	Protein arginine methyltransferase 1
ROS	Reactive oxygen species
SASP	Senescence-associated secretory phenotype
SESN3	Sestrin 3
SIRT1	Sirtuin 1
SKP2	S-phase kinase-associated protein 2
SOD2	Superoxide dismutase 2
TAD	Transactivation domain
TNPO1	Transportin-1
USP7/HAUSP	Ubiquitin-specific protease 7/herpesvirus-associated ubiquitin-specific protease
