# Supplementary material for: FOXO4 as a Redox-Sensitive Regulator of Antioxidant Defense and Cellular Senescence: Cysteine-Based Signaling, p53 Interaction, and Therapeutic Targeting
Source: Antioxidants (Basel). 2026 Jul 3;15(7):842. doi: 10.3390/antiox15070842 (PMC13404206; doi:10.3390/antiox15070842)
Supplement: Supplementary file 1 [file antioxidants-15-00842-s001.zip › antioxidants-4404696-supplementary.pdf]

Supplementary Materials

Table S1. Literature search and selection flow

Selection step	Number	Operational detail
Records assembled from PubMed/MEDLINE, Scopus, and Web of Science	420	Database inception to May 2026; targeted FOXO4/redox/senescence/NRF2 searches
Duplicates removed	120	Deduplication across database exports
Unique records screened by title and abstract	300	Two independent reviewers
Records excluded at title/abstract stage	190	Outside the predefined FOXO4/redox/senescence/therapeutic questions
Full texts assessed	110	Direct FOXO4 relevance or conserved FOXO mechanism potentially applicable to FOXO4
Full texts excluded	21	Incidental FOXO4 mention, unrelated FOXO function, or no relevant mechanistic/translational contribution
Publications retained for qualitative synthesis	89	Included mechanistic, structural, translational, disease-context, and comparator literature

Google Scholar was used exclusively for backward and forward citation tracking and record verification. Because platform-level result counts are unstable and non-reproducible, Google Scholar results were not included as a separate numerical source in the selection flow.

Table S2. Reference-level evidence classification

Category	*n*	Reference numbers in the main manuscript	Use in interpretation
Direct FOXO4-specific	18	19, 21–23, 25–28, 32–34, 42, 44, 46, 64, 65, 69, 81	Permits FOXO4-specific mechanistic or preclinical conclusions.
FOXO-family / conserved	24	6–10, 17, 20, 24, 29, 30, 35–38, 41, 43, 45, 47, 49, 50, 55, 56, 58, 71	Supportive but does not establish that an effect is unique to FOXO4.
Extrapolated predominantly from FOXO1/FOXO3/DAF-16	20	11–16, 18, 31, 39, 40, 51–54, 57, 59, 60, 72–74	Hypothesis-generating only; phrased cautiously and flagged for FOXO4-specific validation.
Contextual redox, senescence, disease, clinical senolysis, or NRF2 literature	27	1–5, 48, 61–63, 66–68, 70, 75–80, 82–89	Provides background or comparison and is not used to infer FOXO4-specific causality.
Total	89	All references classified once	Reference-level categories are mutually exclusive for accounting purposes.

Table S3. Primary sources supporting key FOXO4-specific and translational claims

Claim	Primary reference(s)	Evidence level	Interpretive boundary
Solution structure of the human AFX/FOXO4 DNA-binding domain	[23]	Direct structural	Supports the winged-helix architecture based on the original solution-structure study.
Cys477-dependent FOXO4–p300/CBP redox interaction and acetylation	[19]	Direct biochemical	Supports direct cysteine-based redox sensing by FOXO4.
Cys239/Cys355-dependent FOXO4–transportin-1 interaction and nuclear import	[21]	Direct biochemical/cellular	Supports redox-dependent nuclear trafficking.
FOXO4–p53-dependent senescent-cell survival and FOXO4-DRI senolysis	[22]	Direct cellular/animal	Preclinical; does not establish human efficacy.
Structural and biophysical characterization of FOXO4–p53 and FOXO4-DRI–p53 interactions	[28,64]	Direct structural/biophysical	Defines interaction surfaces and affinity effects; remains preclinical.
FOXO4-DRI in senescent Leydig cells	[65]	Direct preclinical	Tissue-specific animal evidence only.
FOXO4-DRI in senescent vascular endothelial cells and aged mouse aorta	[81]	Direct preclinical	Supports vascular endothelial relevance; does not establish human vascular or cerebrovascular efficacy.
Dasatinib plus quercetin in humans	[68]	Clinical evidence for general senolysis	Provides clinical evidence for senolysis generally but does not constitute clinical validation of FOXO4 targeting.
